# Dynamic Thiol-Disulfide Homeostasis Post-COVID-19 Depends on Age, Gender, and Symptom Severity

**DOI:** 10.7759/cureus.72097

**Published:** 2024-10-22

**Authors:** Tuba Özgöçer, Hakim Çelik, Mehmet R Ceylan

**Affiliations:** 1 Physiology, Harran University, Şanlıurfa, TUR; 2 Infectious Diseases, Harran University, Şanlıurfa, TUR

**Keywords:** age, body mass index (bmi), covid-19, gender, thiol-disulfide homeostasis

## Abstract

Introduction: It has been indicated that the thiol-disulfide homeostasis plays a role in the pathogenesis of COVID-19 infection. We assessed the impact on the thiol-disulfide homeostasis at 15‐day intervals until 60 days, implicated in the pathogenesis of COVID-19, and its clinical relevance in disease progression.

Methods: In this study, 43 COVID-19 patients (18 females and 25 males) were categorized based on symptom severity, age group, and body mass index. Serum samples were collected on days 15, 30, 45, and 60 after COVID-19 diagnosis. Thiol and disulfide parameters were measured in the collected serum samples using spectrophotometric methods.

Results: Serum thiol levels were higher in females and disulfide levels in males (p<0.05). Disulfide levels increased in those older on 15-day post-symptom onset (p<0.05). Serum native thiol levels were higher in patients with moderate and severe symptom severity (p<0.05) than in those with mild severity. The symptoms of chest pain, shortness of breath, loss of taste, and loss of appetite were negatively correlated with thiol levels (p<0.05).

Conclusions: This study suggested critical findings of higher disulfide levels in older age and men, even in the weeks after disease onset. This discovery is significant as it could pave the way for interventions to repair thiol-disulfide homeostasis, potentially transforming the treatment of this group. Moreover, native thiols can point to disease severity even weeks after the onset of symptoms.

## Introduction

The emergence of the severe acute respiratory syndrome coronavirus 2 (SARS-CoV-2) poses a significant global challenge to public health worldwide [1]. Infection with SARS-CoV-2 gives rise to a wide range of clinical presentations, spanning from mild symptoms to severe complications such as acute respiratory distress syndrome, pneumonia, and, in severe cases, multiple organ failures, which are associated with elevated rates of morbidity and mortality [2].

The pathophysiological significance of thiol-disulfide homeostasis (TDH) has been underscored by studies examining inflammation, oxidative stress, and DNA damage in individuals inflicted with COVID-19 [3-8]. Thiols exhibit a high reactivity with free radicals owing to their structural characteristics, leading to the formation of disulfide bonds through oxidation. This oxidative process results in the reversible and reducible nature of these reactions. Consequently, the continual interconversion between thiols and disulfide bonds plays a pivotal role in preserving the intracellular redox environment [9]. The disruption of TDH has been observed in COVID-19 patients, with a decrease in total thiol (TT) and native thiol (NT) levels in severe cases [6,7]. Research has indicated that the conversion of thiols to disulfides via oxidative stress might increase the binding affinity of SARS-CoV and SARS-CoV-2 spike proteins to the ACE2 receptor. This alteration in binding affinity may contribute to enhancing the severity of COVID-19 infection [6]. Thiol-based reducing agents have been discovered to demonstrate antiviral activity against SARS-CoV-2 by directly reducing disulfides on redox-sensitive target proteins essential for viral entry and by modulating the extracellular redox poise required for SARS-CoV-2 entry into cells [5]. Moreover, according to research, thiol-disulfide balance is notably disrupted in patients with COVID-19, suggesting that it could be used as a biomarker for the disease [3,10]. Furthermore, the severity of COVID-19 infection consequences has been associated with the age-dependent reduction of the extracellular thiol-disulfide balance, especially in inflammatory and premature aging disorders [11]. 

This study investigated TDH in COVID-19 patients until the 60th day post-symptom onset (PSO). Our aims are (1) to investigate post-symptom thiol-disulfide levels with a longer-term (PSO 60-day) approach and (2) to evaluate with a different perspective gender, symptom severity, age group, and body mass index (BMI) factors that may lead to differences in TDH. As a secondary aim, we investigated the relationships of some specific symptoms with TDH. Regarding this target, we hypothesized that NTs, which have antioxidant activity, would suppress disease symptoms and that thiol-containing supplements could be a target potential in treatment approaches against the damage caused by COVID-19. Finally, we predicted that adverse clinical findings in male patients may be associated with oxidized disulfide levels.

## Materials and methods

Study design

The study protocol was approved by the Ministry of Health and the Harran University Faculty of Medicine Clinical Research Ethics Committee on May 23, 2022 (approval number: HRU/22.10.16). Written informed consent was obtained from the participants. The inclusion criteria were male and female patients, age 18 and older, with a clinical diagnosis of COVID-19, and those who had not received the COVID-19 vaccination. Exclusion criteria were any special diet and pregnancy. Patients of Turkish ethnicity who applied to the infectious diseases outpatient clinic of Harran University Hospital were included in the study. The study lasted six months (from July to December 2022). COVID-19 diagnosis was confirmed through SARS-CoV-2 reverse-transcriptase polymerase chain reaction (RT-PCR) testing (n=40) or clinical observation (n=3). Nasopharyngeal swabs were collected using standard procedures, and the presence of SARS-CoV-2 was determined by RT-PCR testing (Viracor, Eurofins Clinical Diagnostics, Lenexa, Kansas, United States).

Demographic characteristics, including gender, age, BMI, symptom severity, and COVID-19-related symptoms, were documented through a questionnaire. The COVID-19-related symptoms (cough, fever, shortness of breath, headache, sweating, abdominal pain, loss of appetite) for each participant were documented. Participants were categorized based on symptom grade, age, and BMI. Symptom severity was classified as (1) mild=no additional intervention at home; (2) moderate=medical treatment required; and (3) severe=hospitalization. These data were collected through one-on-one interviews with the patients by a specialist physician.

Sample collection and processing

Patients diagnosed with COVID-19 were individually interviewed, and the symptom onset dates were documented. The day of symptom onset was designated as day 0. Peripheral venous blood samples were obtained from the patients at 15, 30, 45, and 60 days PSO. Serum samples were obtained by centrifuging the collected blood samples at 1500×g for 10 minutes, followed by storage at -80°C until the analysis of thiol and disulfide.

Serum TDH levels were measured using the spectrophotometric method developed by Erel and Neselioglu [12]. Firstly, the levels of NTs in the serum samples were measured by reacting them with 5,5'-dithiobis-2-nitrobenzoic acid (DTNB) without prior processing. Subsequently, the levels of TTs were measured by reducing the dynamic disulfide bonds in the serum samples using sodium borohydride (NaBH4) to generate free functional thiol groups. Formaldehyde was then used to remove any unused NaBH4 completely, and the TT groups, including both reduced and NT groups, were measured by reacting the samples with DTNB. Since the reduction of a disulfide bond generates two distinct thiol groups, the amount of dynamic disulfide (SS) bonds was calculated by determining the 50% difference between the TT and NT groups. The ratios of SS/NT, SS/TT, and NT/TT were also calculated. Subsequently, the disulfide (Ds) concentration was calculated using the formula: Ds concentration=(TT concentration−NT concentration)/2. Furthermore, the Ds/TT (%), Ds/TT (%), and NT/TT (%) ratios were computed based on the previously measured NT and TT levels [12]. TDH parameters and their ratios at 15, 30, 45, and 60 days PSO were defined as 1, 2, 3, and 4, respectively.

Statistical analysis

Statistical analysis was conducted using IBM SPSS Statistics for Windows, Version 25.0 (Released 2017; IBM Corp., Armonk, New York, United States). The Shapiro-Wilk test was employed to assess the normal distribution of the data. Data that exhibited a normal distribution were presented as mean±standard error-repeated measures. Numbers (n) and percentages (%) were used to represent categorical variables (percent). In parametric assumptions, the repeated-measures analysis of variance (ANOVA) test was used for the statistical analysis of dependent variables measured at four different times (15, 30, 45, and 60 days PSO). The paired-samples t-test was used to investigate the relationship between two dependent variables. For non-parametric assumptions, the Mann-Whitney U test was used to compare independent groups (age, gender, symptom grade). Spearman's correlation coefficient was utilized to analyze correlations between parameters. A significance level of p<0.05 was considered statistically significant.

## Results

Profile of patients

A total of 43 COVID-19 patients, 18 women (41.9%) and 25 men (58.1%), were included in our study. Table 1 summarizes the gender, symptom grade, COVID-19-related symptoms, age groups, and BMI classes of all participants. All participants included in the study were of Turkish ethnicity, with a median age of 40 years (range: 19-57).

**Table 1:** Demographic and clinical characteristics of COVID-19 patients. BMI: body mass index

COVID-19 clinical characteristics	(n)
Total number of patients	43
Symptomatic	43
Non-symptomatic	0
Overall symptom grade	(n)
1 (mild)	5
2 (moderate)	21
3 (severe)	17
COVID-19-related symptoms	(n)
Cough	34 (79%)
Fever	37 (86%)
Shortness of breath	35 (81.3%)
Headache	29 (67.4%)
Sweating	29 (67.4%)
Abdominal pain	21 (48.8%)
Loss of appetite	26 (60.4%)
Male gender (%)	25 (58.1%)
Age classes	(n)
Age 1 (≤36)	19
Age 2 (37-46)	13
Age 3 (47-57)	11
BMI classes	(n)
BMI 1 (normal)	18
BMI 2 (overweight)	17
BMI 3 (type 1 obese)	6
BMI 4 (type 2 obese)	2

TDH parameters according to PSO days, gender, symptom grade, age, and BMI groups

TDH levels exhibit a striking degree of variability during the initial onset of symptoms and follow-up to the 60th day, further influenced by factors such as gender, symptom grade, age, and BMI.

**Figure 1:**
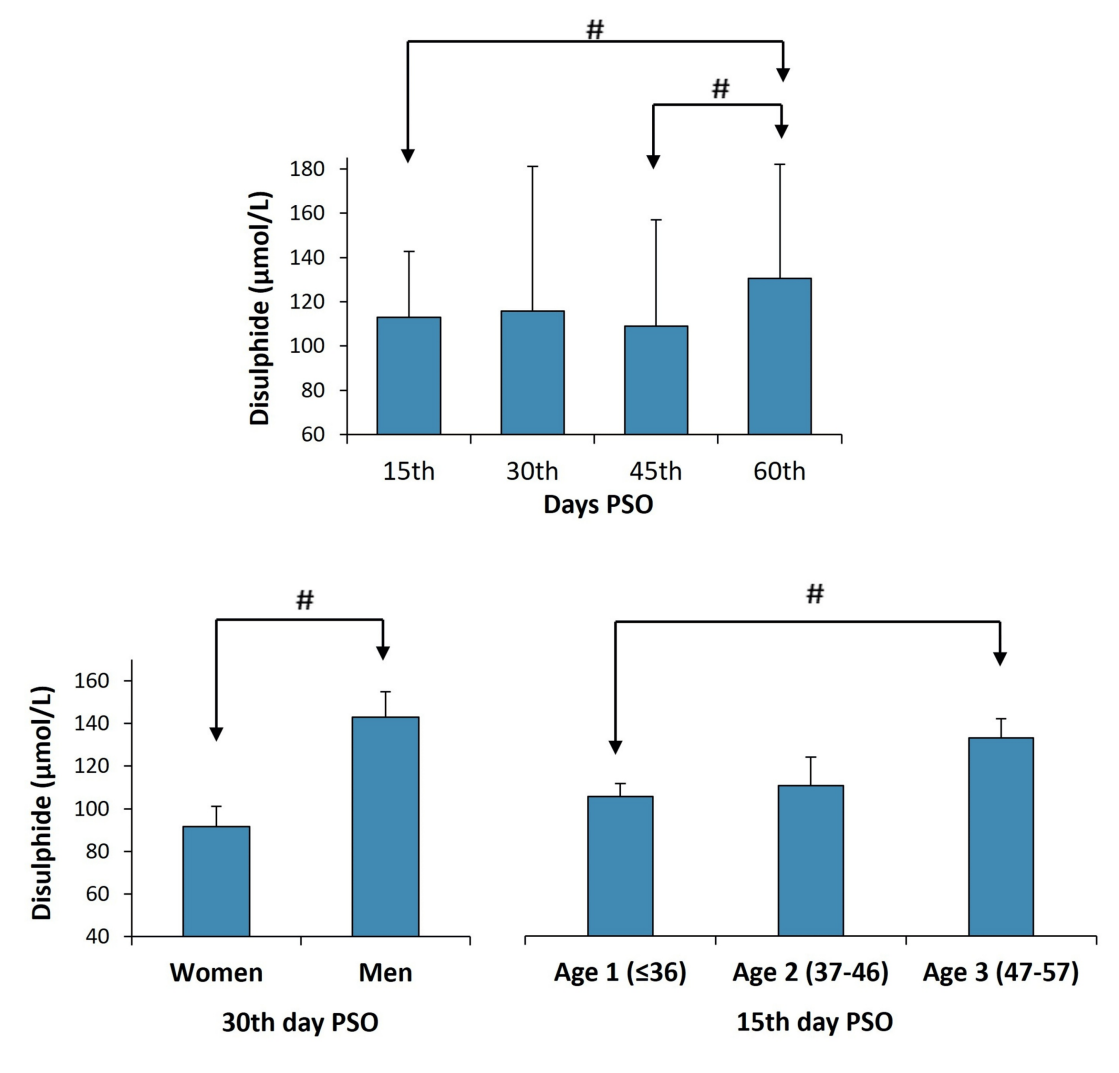
Disulfide levels (µmol/L) according to days post-symptoms and groups. The paired-samples t-test was used to compare PSO days. The Mann-Whitney U test was used to compare groups of age and gender. p<0.05 was considered statistically significant. #p<0.05 PSO: post-symptom onset

Disulfide levels were higher on the 60th day than on the 45th and 15th days PSO (p<0.02) (Table 2, Figure 1).

**Table 2:** Thiol-disulfide homeostasis parameters (mean±SEM) differ according to PSO days. 15th, 30th, 45th, and 60th days PSO were defined as 1, 2, 3, and 4, respectively. The paired-samples t-test was used. p<0.05 was considered statistically significant. #Compared with 15th and 45th days PSO (p<0.05). *Compared with 45th day PSO (p<0.05). NT: native thiol; TT: total thiol; SS: disulfide; PSO: post-symptom onset

	15th day PSO (1)	30th day PSO (2)	45th day PSO (3)	60th day PSO (4)
NT (µmol/L)	322.0±83.2	334.1±144.2	324.7±96.6	295.2±87.2
TT (µmol/L)	552.6±75.1	575.8±101.8	536.2±113.6	581.9±134.4
SS (µmol/L)	113.0±29.7	115.9±65.3	109.0±48.1	130.6±51.4^#^
SS/NT (%)	37.3±18.9	39.8±35.7	34.3±20.0	41.1±21.7
SS/TT (%)	20.8±0.9	21.6±1.5	19.7±1.1	23.8±0.9^*^
NT/TT (%)	58.3±1.9	56.7±3.1	60.5±2.3	52.3±1.9^*^

The SS 2, SS/NT 2, and SS/TT 2 levels were higher in men than in women; these differences were statistically significant (p=0.003, p=0.013, and p=0.013, respectively), while NT/TT 2 level was lower in men than in women (p=0.013) (Figure 1).

The disulfide level on the 15th day PSO was more significant in age 3 participants (133.00±8.92) compared to age 1 (105.49±6.11) groups (p<0.05) (Figure 1). No difference was observed according to age groups for other TDH parameter levels (p>0.05).

**Figure 2:**
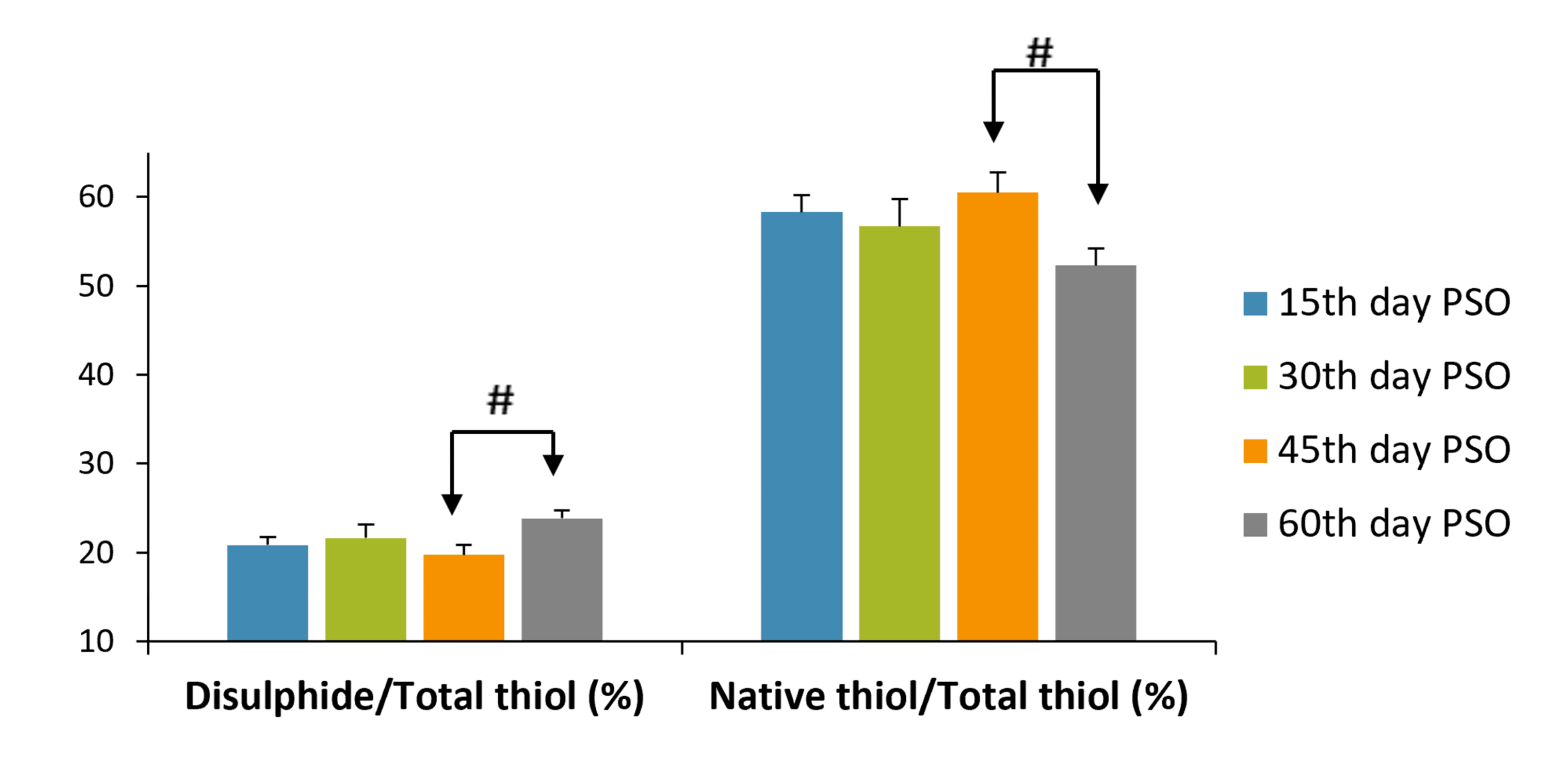
Native thiol (µmol/L), total thiol (µmol/L), disulfide/total thiol (%), and native thiol/total thiol (%) levels according to days post-symptoms. The paired-samples t-test was used to compare PSO days. p<0.05 was considered statistically significant. #p<0.05 PSO: post-symptom onset

SS/TT levels were higher on the 60th day than on the 45th day PSO (p<0.02), while NN/TT levels on the 60th day PSO were lower than the 45th day PSO (p<0.05) (Table 2, Figure 2). There was no significant difference in NT, TT, and SS/NT levels (p>0.05) compared to PSO days (Table 2).

**Figure 3:**
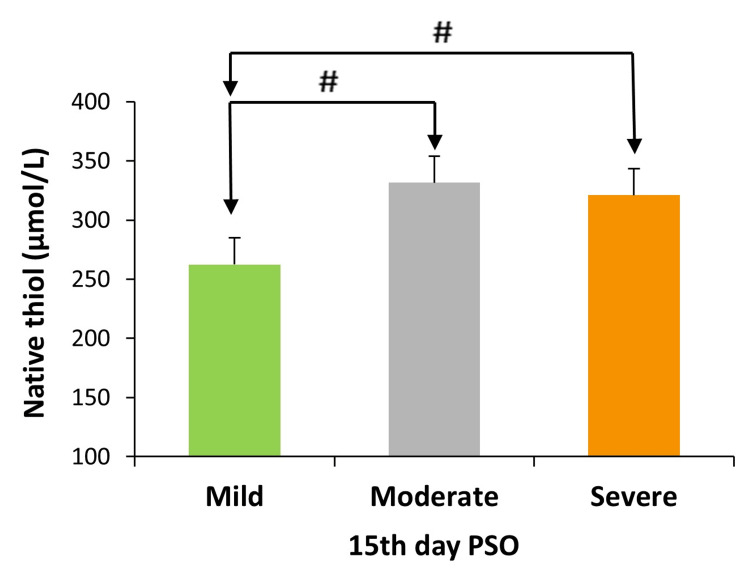
Native thiol levels (µmol/L) based on the severity of symptoms on the 15th day PSO. The Mann-Whitney U test was used to compare groups of symptom grades. p<0.05 was considered statistically significant. #p<0.05 PSO: post-symptom onset

Mild (n=5), moderate (n=21), and severe (n=17) symptom groups were compared according to overall symptom grade, and NT levels (on the 15th day PSO) in the mild group (262.46±22.37) were lower than moderate (331.51±19.26) and severe groups (320.93±23.53) (p<0.05) (Figure 3). No significant differences (p>0.05) were observed in the levels of other TDH parameters when compared across symptom groups.

The comparison between the normal-weight (n=18) and overweight (n=17) groups was conducted based on BMI classifications (Table 1). There was no significant difference between the groups in TT, NT, and SS levels (p>0.05). However, SS/NT 1 and SS/TT 1 levels were higher in BMI 1 than BMI 2; these differences (43.37±4.37 vs 35.82±5.35 and 22.26±1.63 vs 19.72±1.29, respectively) were statistically significant (p=0.03). Additionally, NT/TT 1 levels were statistically significantly lower in BMI 1 (55.47±3.26) than in BMI 2 (60.55±2.58) (p=0.03).

Correlation of COVID-19-related symptoms and NT, TT, and SS levels

A negative correlation was observed between the NT 1 and SS 1 (rho=-0.544; p<0.001) and SS 2 (rho=-0.341; p=0.02). However, NT 2 level with chest pain symptom (rho=-0.540; p=0.04), NT 1 level with loss of appetite symptom (rho=-0.563; p=0.02) and loss of taste symptom (rho=-0.589; p<0.003), and TT 1 level with shortness of breath symptom (rho=-0.509; p=0.019) were significantly negatively correlated. Surprisingly, SS 2 level with weakness symptom (rho=-0.419; p=0.03) and SS 3 level with weakness symptom (rho=-0.424; p=0.04) were significantly negatively correlated, while SS 3 level with loss of smell symptom (rho=0.505; p=0.02) was significantly positively correlated (Table 3).

**Table 3:** Correlation between COVID-19-related symptoms and NT, TT, and SS levels. These levels are defined as 1, 2, 3, and 4, corresponding to the 15th, 30th, 45th, and 60th days PSO. Spearman's correlation analysis *p<0.05 was considered statistically significant. NT: native thiol; TT: total thiol; SS: disulfide; PSO: post-symptom onset

		NT1	NT2	NT3	NT4	TT1	TT2	TT3	TT4	SS1	SS2	SS3	SS4
Chest pain	rho	0.208	-0.540*	-0.389	0.502	0.069	-0.481	-0.481	0.671*	-0.224	-0.102	-0.219	0.265
	P-value	0.475	0.046	0.212	0.096	0.816	0.082	0.114	0.017	0.442	0.729	0.494	0.405
Shortness of breath	rho	-0.372	-0.062	-0.322	0.119	-0.509*	0.270	-0.244	0.277	-0.163	0.299	0.109	0.327
	P-value	0.096	0.789	0.192	0.638	0.019	0.237	0.330	0.266	0.479	0.189	0.667	0.186
Weakness	rho	0.028	0.254	0.053	-0.086	-0.197	-0.296	-0.233	-0.077	-0.082	-0.419*	-0.424*	-0.022
	P-value	0.890	0.210	0.809	0.689	0.325	0.142	0.284	0.721	0.683	0.033	0.044	0.920
Loss of taste	rho	-0.589*	-0.098	-0.049	0.064	-0.307	-0.152	-0.059	0.112	0.124	-0.025	0.006	0.006
	P-value	0.003	0.655	0.827	0.784	0.154	0.490	0.793	0.630	0.574	0.910	0.978	0.980
Loss of smell	rho	-0.237	-0.118	-0.047	-0.091	-0.210	0.025	0.354	-0.122	0.024	0.103	0.505*	-0.150
	P-value	0.289	0.601	0.845	0.703	0.348	0.913	0.125	0.609	0.916	0.649	0.023	0.527
Loss of appetite	rho	-0.563*	-0.064	0.211	-0.178	-0.239	-0.051	0.114	-0.357	0.396	0.181	0.038	-0.157
	P-value	0.029	0.828	0.449	0.542	0.390	0.863	0.686	0.210	0.144	0.536	0.894	0.593

## Discussion

This study followed COVID-19 patients regarding TDH and potential associations with various factors, including days PSO, gender, age, BMI, and symptom grade. The current study offers a different approach by considering the relationships between disease symptoms and TDH parameters separately. It also has the potential to contribute to therapeutic searches by monitoring patients for two months after symptoms and evaluating the changes in thiol-disulfide levels with sub-factors.

Alterations in TDH occur in the days following symptom onset

The occurrence of post-COVID-19 symptoms received considerable attention, as a substantial percentage of individuals have reported developing protracted and late-onset symptoms after the acute phase of the infection [13-16]. These symptoms have been systematically assessed, with prevalence reported at various time points post-onset, indicating the persistence of symptoms beyond the acute phase of the disease [13,16]. Considering this situation, it is predicted that short-term and longer-term investigations of TDH are needed. The current study is the first study to evaluate TDH in COVID-19 patients with the prolonged and late-onset symptoms approach. Our findings revealed a notable elevation in the SS levels and the SS/TT ratio on day 60 PSO. Furthermore, we observed that the NT/TT ratio decreased on day 60 PSO. These findings indicate persistent and prolonged endogenous antioxidant fluctuations within 60 days PSO. During the acute phase (first 24 hours) of COVID-19 infection, NT and disulfide concentrations decreased in the severe patient group relative to the control and mild patient groups, according to short-term clinical studies [3]. Recently, Bektemur et al. reported that individuals infected with COVID-19 exhibited elevated concentrations of disulfide, while TT and NT levels decreased in comparison to those of healthy individuals [17]. Although the aim of our study is not to compare with the control group, it is observed that there are fluctuations in TDH parameters in the follow-up days after the onset of symptoms and there is an increase in SS and SS/TT levels on the 60th day. These findings may be associated with prolonged or late-onset symptoms after infection. In viral respiratory infections such as COVID-19, symptoms and laboratory results are more apparent in the first two weeks. As this study shows, the first month of the disease is probably more decisive on TDH parameters.

The TDH is associated with the symptoms

We analyzed the level of TDH in 43 patients presenting with mild, moderate, and severe symptoms on serial day 60 PSO. Accordingly, NT1 levels were initially higher in the moderate and severe symptom groups on the 15th day PSO, but these differences diminished in subsequent evaluations. Endogenous antioxidant molecules (NT and TT) are physiological components that reduce the harmful impacts of reactive oxygen species (ROS). ROS generated in the body interacts with the sulfhydryl groups (-SS) of native antioxidants, converting them into disulfides. Additionally, in the current study, the negative correlation observed between symptom duration (chest pain, loss of taste, loss of appetite) and NT1 levels supports the potential utility of thiol derivatives as supplements in disease treatment. There is a significant negative correlation between TT1 level and shortness of breath, while SS2 and SS3 levels have a negative correlation with weakness. Additionally, SS3 level has a positive correlation with loss of smell. There are studies on using herbal products in managing COVID-19 patients and complementary treatment approaches in alternative medicine [18]. Several studies have highlighted the potential role of thiols [19], a balanced diet and use of supplementation [20], and vitamin D [21] in the treatment and management of COVID-19. Cazzola et al. suggested that thiol-based drugs, such as N-acetylcysteine and glutathione, may represent a novel therapeutic approach to address the cytokine storm syndrome and respiratory distress observed in COVID-19 pneumonia patients. Furthermore, thiol concentrations were found to predict disease severity in COVID-19 patients compared to healthy controls [8]. Similarly, Ducastel et al. observed that thiol concentrations decreased with disease severity in COVID-19 patients and were associated with increased oxygen needs and intensive care unit admission [22]. These findings underscore the potential significance of thiols as biomarkers and their association with disease severity in COVID-19. Moreover, the disruption of thiol-disulfide balance in COVID-19 patients has been noted in several studies [3,10]. This disturbance has been proposed to hold predictive value in diagnosing COVID-19, determining its severity, and establishing its correlation with the presence and duration of symptoms. Furthermore, elevated thiol concentrations or antioxidant capability have been connected to the possible impacts of TDH on clinical development and prognosis in COVID-19 [23]. In conclusion, according to our study's findings, we predict a negative relationship between thiol and the symptoms of chest pain, shortness of breath, loss of taste and appetite, and thiol-based supplements may affect the treatment and prognosis of COVID-19. However, additional research, encompassing controlled clinical trials, is required to substantiate the potential anti-COVID-19 mechanisms and properties of thiols and ascertain their exact role in managing COVID-19.

The TDH is negatively influenced by male gender

In this study, the increase in disulfide levels in male patients on the 15th day of PSO indicates that gender affects TDH. This discovery underscores the necessity of a gender-based approach in treatment interventions by highlighting the presence of potential gender-specific differences in the regulation of oxidative stress and cellular function following COVID-19. Although the available references do not explicitly address the direct comparison of thiol-disulfide levels between genders, the evidence suggests the potential existence of gender-specific differences in TDH across various diseases and conditions [23]. Gebhard et al. point out the impact of innate and adaptive immune responses based on the sex hormone of the ACE2 receptor, gender-specific lifestyle, health behavior, psychological stress, and socioeconomic conditions on COVID-19 [24]. Lately, Hammo et al. found a higher oxidative stress response in male rats in their study examining the role of gender in protection against doxorubicin-induced oxidative stress [25]. In this particular context, conducting additional research that specifically examines the potential influence of gender on oxidative stress within COVID-19 would be of great significance in comprehending the intricate relationships between gender, oxidative stress, and disease outcomes.

The TDH is age- and BMI-dependent

The current study demonstrates that TDH may be influenced by BMI and age differences, with higher disulfide levels observed in patients over 47 years of age compared to patients under 36 on the 15th PSO. The impact of age on this pandemic disease is evident in various aspects, including susceptibility to infection and, more significantly, the severity of symptoms, mortality, and disability [26]. Indeed, only a tiny number of individuals under the age of 30 encounter significant illness, while the majority stay asymptomatic or show mild symptoms. Furthermore, the mortality rate is exceedingly low among the youngest age groups [26,27]. Our study detected a decrease with age in the homeostatic mechanisms governing thiol redox and an increase in disulfide levels. This finding indicates that these changes contribute to causal events and serve as a molecular indicator of increased risk of infection and the onset of severe disease. Moreover, the study found that in overweight patients compared to normal-weight patients, the SS/NT ratio was lower due to the relative increase in native thiol levels. In comparison, the NT/TT ratio was higher. Multiple studies have provided evidence supporting a substantial correlation between elevated BMI and negative results related to COVID-19 [28,29]. Similarly, an age-dependent effect of BMI ≥30 kg/m^2^ on COVID-19 severity or death among adults was reported [29,30]. We think the difference in overweight patients in our study may be related to the diet containing native thiols with antioxidant properties. Even so, it is imperative to consider the possible effects of multiple factors, such as comorbidities and differences between regions, on the severity of COVID-19.

The present study is subject to several limitations. Firstly, the patient sample size was relatively small due to the practice of serial sampling. Secondly, the study design did not include a control group for comparative evaluation. Because our study focused on investigating the lasting effects of TDH, we didn't assess the impact of ROS activity during the acute phase.

## Conclusions

The current study researching the role of thiols and oxidative stress in post-COVID-19 symptoms may contribute to developing targeted interventions to manage and alleviate long-lasting and late-onset symptoms in COVID-19 survivors. According to our study, it appears possible to have COVID-19-specific symptoms by increasing NT levels. Our data also indicated that older age and male gender are disadvantaged due to high disulfide levels. Further investigation is warranted to understand the underlying mechanisms and potential therapeutic implications of the association between post-COVID-19 symptoms, oxidative stress, and TDH.
